# Provenance information as a tool for addressing engineered nanoparticle reproducibility challenges

**DOI:** 10.1116/1.4964867

**Published:** 2016-10-19

**Authors:** Donald R. Baer, Prabhakaran Munusamy, Brian D. Thrall

**Affiliations:** Earth and Biological Sciences Directorate, Pacific Northwest National Laboratory, Richland, Washington 99352

## Abstract

Nanoparticles of various types are of increasing research and technological importance in biological and other applications. Difficulties in the production and delivery of nanoparticles with consistent and well defined properties appear in many forms and have a variety of causes. Among several issues are those associated with incomplete information about the history of particles involved in research studies, including the synthesis method, sample history after synthesis, including time and nature of storage, and the detailed nature of any sample processing or modification. In addition, the tendency of particles to change with time or environmental condition suggests that the time between analysis and application is important and some type of consistency or verification process can be important. The essential history of a set of particles can be identified as *provenance information* and tells the origin or source of a batch of nano-objects along with information related to handling and any changes that may have taken place since it was originated. A record of sample provenance information for a set of particles can play a useful role in identifying some of the sources and decreasing the extent of particle variability and the lack of reproducibility observed by many researchers.

## I. INTRODUCTION

Nanoparticles or more generally nano-objects^1^ are finding increasing use in many areas including applications in biological systems such as agents for drug delivery, antioxidants, contrast agents for imaging, sensing, and for assisting understanding of basic biological processes.^2–4^ In addition to beneficial effects, there is concern about possible negative impacts and toxicity.^5–8^ Unfortunately, difficulties associated with the consistent and reproducible production and delivery of nano-objects challenge both their productive application and appropriate understanding of any deleterious effects. Nanomaterial consistency issues have raised the concerns of many researchers around the world.^7,9–18^

There has been much discussion in the literature about characterization needs for nanoparticles and other nanomaterials,^14,18–23^ including long lists of desired measurements that would be prohibitively costly and might not actually help improve the material reproducibility. Although it is generally agreed that many studies using nanomaterials have not collected or reported sufficient information about the materials used to enable experiments to be reproduced,^14,15^ the inherent nature of nano-objects suggests that characterization alone may not adequately address some of the reproducibility issues. One of the central questions to be addressed concerns the nature and type of information needed so that a user or researcher can trust that the materials being used or tested have the desired or intended properties.^24^ As noted below, “provenance” is a term that is being increasingly used to describe issues of data and information reliability.

Provenance is most commonly used in relation to the origin of a work of art. However, the use of provenance is being extended to other areas^25^ with one working group having generalized the concept as follows: Provenance of a resource is a record that describes entities and processes involved in producing and delivering or otherwise influencing that resource. Provenance provides a critical foundation for assessing authenticity, enabling trust, and allowing reproducibility.^26^ In dealing with nano-objects, we are in effect concerned with the provenance of the material.

It is necessary to ask what information is needed to enable trust and reproducibility for nano-objects. To help identify an answer, it may be useful to adapt an ISO description of provenance information: Information that documents the history of [a batch of nano-objects]: This information tells the origin or source of the [batch of nano-objects], any changes that may have taken place since it was originated, and who has had custody of it since it was originated. Examples of provenance information are the principal investigator who recorded the data, and the information concerning its storage, handling, and migration [adapted from ISO/TS 13527:2010, 1.4.2.36 where the terms in square brackets have been altered from the ISO definition]. The intent and objectives of provenance information are clear, as is the importance of origin and history. The description highlights the need for both the collection and retention of relevant provenance information and some type of data record associated with a set of nano-objects. Our concern is the range of information that needs to be included as provenance information in some type of information and data record. This perspective addresses some aspects of data completeness, but does not specifically address important issues and opportunities related to data collection and storage frameworks that allow information to be mined and properties such as risk modeling,^27–29^ data curation,^24^ or statistical issues in data or sample replicability.^30^

## II. PROVENANCE INFORMATION AND DATA RECORDS FOR NANO-OBJECTS

The remainder of this article explores the nature of provenance information that would be useful and relevant to associate with a batch of nano-objects in a data record that travels with or is associated with the material. The objective is not to define a specific set of information or data requirements because the requirements can depend on the material and/or application. It is useful to think about a framework or context for collecting and reporting provenance information that relates to sample consistency and reproducibility. Three considerations can help guide our thinking as summarized in an *ACS Nano* editorial about nanomaterials characterization:^14^

(1) Characterization requirements vary with material and application: Consistent with the stated nanoparticle characterization needs applied for submissions to the journal *ACS Nano*,^14^ appropriate provenance information should be based on the nature and use of the nano-objects, knowledge of the relevant physicochemical characteristics for the material, and understanding of the common behaviors of nanomaterials. There is no single useful and ideal list of required measurements.
(2) Intrinsic characteristics: However, as suggested in the editorial and other publications,^18,20^ there are some commonly important parameters that are intrinsic characteristics of a nano-object such as size, size distribution, chemical composition, purity, crystallinity (where appropriate), shape or morphology, surface chemistry and charge (where appropriate), and surface area that are useful for most materials and appropriate to document as part of a nano-object data record.
(3) Acquired or extrinsic characteristics: Characteristics of nano-objects are also acquired or altered during storage, handling, processing, or following suspension in experimental biological or environmental media. These can include hydrodynamic diameter in specific media, size changes due to dissolution, aggregation or agglomeration, surface reactivity (e.g., the redox or membranolytic activity), and charge or zeta potential. Information about the treatments, handling, storage, and altered properties should be included as important components of provenance information in nano-object data records and may be among those most widely ignored or currently under reported.

We have found and reported on several issues that relate to or expand upon the above categories and that impact the behaviors of nanoparticles. These include details of synthesis,^31–33^ time, handling and storage,^34,35^ and the presence of likely or unexpected contaminants.^10,36^ At a high level, we have grouped issues into three categories: (1) nanoparticles are not (generally) created equal; (2) the high ratio of surface to bulk atoms means that surfaces and interfaces are especially important for nanomaterials; and (3) nanoparticles are dynamic, and they change with time and environment.^10,37^ Recognition of these three general and somewhat overlapping characteristics of nanomaterials helps identify information that can be important for the delivery of reproducible nano-objects that should become part of the provenance information associated with those objects.

### A. Nanoparticles are not usually created equal

Many published papers include simple general descriptors of particles in titles such as the “…behaviors of *element X* (where X might be Ag, Au, SiO_2_, Fe, or almost any element or compound) nanoparticles.” In reality, the behaviors described are usually the properties of a subset of nanoparticles from a batch of particles created by a specific process or sequence of processes. They have also been studied at some time after synthesis, handling, and some type of storage (e.g., in some type of container for specific environmental conditions). In many cases, the properties of these “specific” particles should not be viewed as “generally” applicable. The following examples highlight differences in properties of Fe metal-core oxide-shell particles that, based on paper titles, might have been assumed to have similar properties. In addition to differences in behaviors of particles produced by totally different processes, seemingly minor changes in a synthesis process can produce particles with significantly altered behaviors^33^ and sometimes differences occur in spite of major efforts to reproduce every step of a process in detail.^12^

**Fig. 1.**
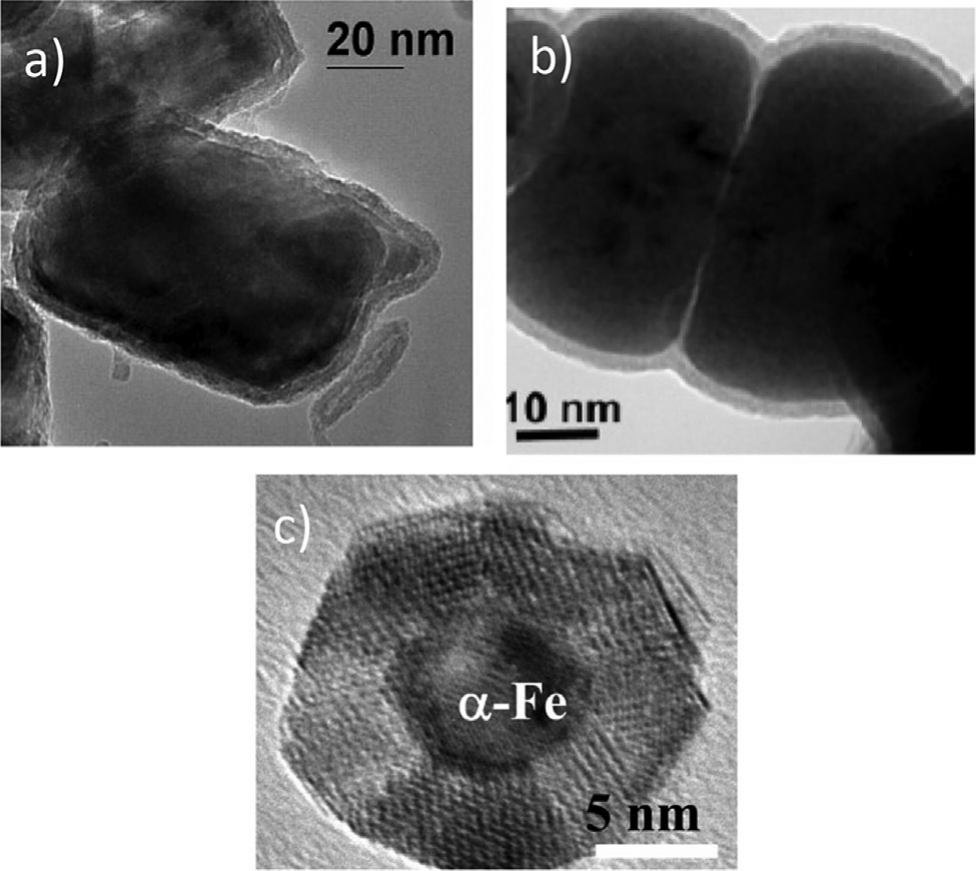
Electron microscopy images of Fe metal-core oxide-shell nanoparticles synthesized by three different processes: (a) hydrogen reduction of oxide (Fe^H2^), (b) reductive precipitation in water (Fe^BH^), (c) sputter gas aggregation (Fe^SP^). Particles (a) and (b) are nominally the same size but have significantly different core and shell structures. Particle (c) is smaller in size but with a structure similar to (a). Each type of particles has different reaction properties with CCl_4_ in water. Adapted from Refs. 31, 35 and 37.

Metal-core oxide-shell Fe nanoparticles have been studied as a way to reduce contaminants in ground water.^38,39^ We found that both reaction rates and reaction pathways vary significantly for nanoparticles produced in different ways.^40^ The reaction of the three types of Fe metal-core oxide-shell nanoparticles shown in Fig. 1 with CCl_4_ in water produced significant differences in reaction rates and products formed. These particles were formed by (1) reduction of goethite and hematite particles with H_2_ at high temperatures (200–600 °C) [Fe^H2^],^41^ (2) reductive precipitation of FeCl_3_ with NaBH_4_ [Fe^BH^],^42^ and (3) sputter gas aggregation [Fe^SP^].^43^ Nurmi *et al*.^31^ found reduction of CCl_4_ in water by Fe^BH^ produced more chloroform (CCl_3_H) than produced by reduction with Fe^H2^, which produced more environmentally benign products. The Fe^BH^ and Fe^H2^ particles were nominally the same diameters (40–60 nm), but otherwise had some fundamentally different characteristics. The metallic cores of the Fe^H2^ particles were made up of nearly single crystal grains essentially the size of the particles surrounded by a highly crystalline oxide shell. In contrast, the metallic cores of the Fe^BH^ particles were made up ≈1 nm grains with this collection of metallic grains surrounded by an oxide shell that did not appear to have high crystallinity. Although not a surprise to many researchers, this example affirms that more than nanoparticle size is required to identify nanoparticle properties or behaviors.

Interestingly, in comparison to the Fe^BH^ and Fe^H2^ nanoparticles, the relatively pure Fe^SP^ particles were observed to oxidize more slowly and were almost unreactive with the CCl_4_.^37^ Although Fe^H2^ and Fe^SP^ were different in size, both contained highly crystalline metal cores and crystalline oxide shells. We postulated that the large difference in corrosion rate and reactivity with CCl_4_ might be related to S retained in the nanoparticles from the salt used in part of the synthesis process.^41^ In a follow up study, Moore *et al*.^36^ examined the impact of a range of anions in precursor salts on the behaviors of Fe^H2^ “type” particles for which the only difference was the nature of the salt used in the synthesis process. The resulting particles had significant variations in both reaction rate and the formation of chloroform. Although only trace residues from the anions present during synthesis remained in the particles, the reaction properties were significantly altered. The particles produced by this process were generally similar to the Fe^H2^ particles described above, but produced a wide range of reactivity and product formation (some similar to the Fe^BH^ particles) and all were more reactive than the Fe^SP^ particles. We have found that both major and relatively subtle differences in nanoparticle synthesis contribute to variations in nanoparticle properties.

*Synthesis and process related provenance information*: The above examples for relatively simple nanoparticles demonstrate that the synthesis approach and synthesis details can have a significant impact on particle properties. Many other examples could be provided,^7,32^ and the challenge of variability increases with the complexity of the nanoparticles such as the addition of coatings with designed or intended functionality.^12^ In an earlier study, we examined the literature related to the benefits and/or toxicity of ceria nanoparticles and found that more than one-third of the papers reporting health effects did not have sufficient information to allow a group of material scientists to assess the method used to produce the particles.^33^

Based on what has been observed, the range of information about particle synthesis appropriate for sample provenance information should include the following:

(1) Record of sample synthesis: reference or details of synthesis as known (e.g., process, vendor, lot number, chemicals, and chemical sources)
(2) Characterization results: data reports including relevant dates and processing of samples for analysis
(3) Important dates and times: synthesis, arrival in laboratory, opening of sample container, primary analysis measurements, and expiry date
(4) Storage time, conditions, and containers: temperature, humidity, media, light shielded, shipping, or transport)
(5) Record of additional processing: e.g., dried, washed, heated, sonicated, functionalized (including the method and number of times processed).

It is relevant to remember that the information noted above is important and may be helpful in understanding differences in particle behaviors in biological systems. However, such information may not always be sufficient to distinguish differences in the biological effects of particles, especially when particles have been stored after characterization. Therefore, some type of system or biologically relevant test is often useful to verify similarity of particle properties for the intended purposes near the time of application. Such tests might include verifying size in media using dynamic light scattering (DLS) or another method. The consistency of effective surface potential might be verified using a zeta potential measurement just before application or use, the surface composition or consistency might be verified by XPS, and it is often important to check for presence of endotoxin or other biological toxicity response on known systems (positive and/or negative control).

### B. Surfaces are important and difficult to control

Although the significance of nano-object surfaces is widely acknowledged and the relationship of surface properties to biological responses is long established,^44–47^ the importance of knowing and understanding the nature of nano-object surfaces seems to be somewhat ignored by some researchers,^8^ and the effort required to create and characterize nano-object surfaces is not fully appreciated.^10,13^ Although particle changes are discussed in Sec. II C, it is important to recognize that surfaces are the boundary between nano-objects and their environments. The nature of surfaces impacts how particles interact with their environment and the environment impacts the surface composition and functional nature of the surface.

Two related issues regarding nano-object surfaces are highlighted as critical to particle reproducibility: (1) Are there unwanted surprises on the surface? (2) Does the surface have the composition or functionality that is intended or desired?

Researchers using surface analysis methods frequently find elements or compounds on surfaces that are not those intended or desired. As described in an earlier paper, F made up of Polytetrafluoroethylene (PTFE) break down products arising from a component in the synthesis system was found on the surfaces of CuOx nanoparticles intended for toxicology studies.^10^ Because of the low overall concentration and the presence primarily at the surface, the likely toxic PTFE break-down layer on the particles would have been undetected without the application of an appropriate surface sensitive analysis method, XPS in that case. Surface analysis methods are very useful in help identifying and minimizing unplanned species that can appear on surfaces.

Bacterial endotoxin is a heat-stable common contaminant in many chemicals and glassware that readily attaches to nanoparticles.^48^ Undetected endotoxin or other bacterial contamination can mask a true understanding of the biological impacts of nanoparticles or provide false-positive indications of the potential of a material to cause biological effects, such as inflammation. As noted in several studies, nanoparticles can also complicate endotoxin detection,^48,49^ and both a high degree of care in the preparation and handling of particles and appropriate testing are required.

**Fig. 2.**
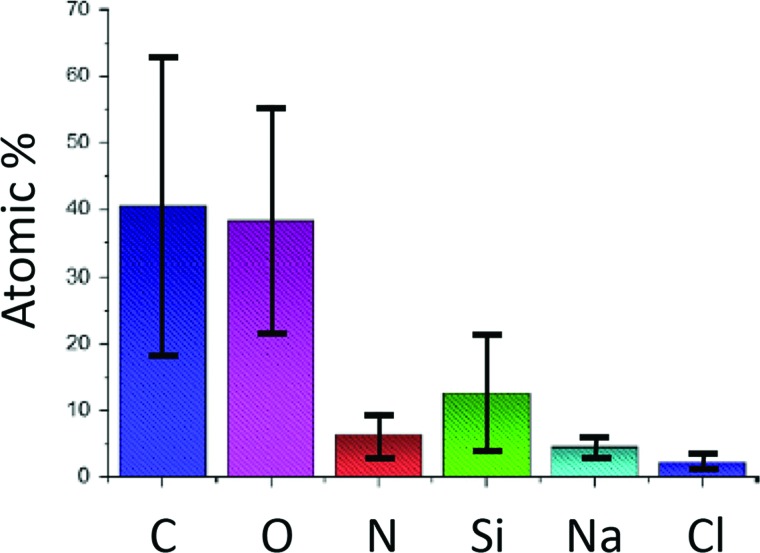
Mean atomic percentages obtained from XPS data and variations among different batches of magnetite@silica nanoparticles prepared with identical procedures. In spite of significant effort, sample reproducibility was low. Adapted from França *et al.*, J. Colloid Interface Sci. **389**, 295. Copyright (2013), Elsevier.

Surface coatings with a designed function are increasingly important to many types of nano-object applications, including drug delivery and other types of functionality. As described in some detail by França *et al*.,^12^ in spite of careful effort, it is not always possible to achieve the desired surface chemistry or coverage. In the words of the authors, “Numerous samples of magnetite@silica and magnetite@silica@silane core–shell nanoparticles were prepared by an experienced chemist, using the same identical equipment and the same lots of reagents.” Their surface analyses showed “batch-to-batch chemical variations: no two batches were found to have the same surface chemistries, showing unexpected Si–O bond scission and amine oxidation.” The compositional variation found on the magnetite@silica particles is shown by the large error bars in Fig. 2. Similar consistency challenges were observed in follow up studies by this same group where batch to batch inconsistency was preparation-independent and effects of careful water washing were observed.^50,51^ Although these types of reproducibility challenges are only infrequently reported in detail,^11^ they are likely to be much more common than reported or even recognized.

*Surface related provenance information*: The relevant question regarding nano-object surfaces concerns what analytical or functional measurements have been conducted to provide researchers or others confidence that the specific nano-objects to be used for a specific purpose or a study have the expected surfaces and surface properties?

Achieving the needed confidence does not necessarily mean conducting a full set of analysis before each application. In the work described above focusing on Fe metal-core oxide-shell nanoparticles, a wide variety of analytical and other measurements were conducted on the material during initial studies.^31^ Once we understood the nature of the particles and the consistent, as well as seemingly inconsistent, information provided by the full range of methods, we narrowed our routine measurements to a much smaller subset of tools that could be usefully applied on a routine basis at a relatively low cost. For example, in electrochemical tests, one type of particle processed in a specific way had nicely reproducible open circuit potentials. Thus, based on established information, it was possible to verify one type of functional consistency for each use of different samples from a larger batch of characterized material.^31,52^

**Fig. 3.**
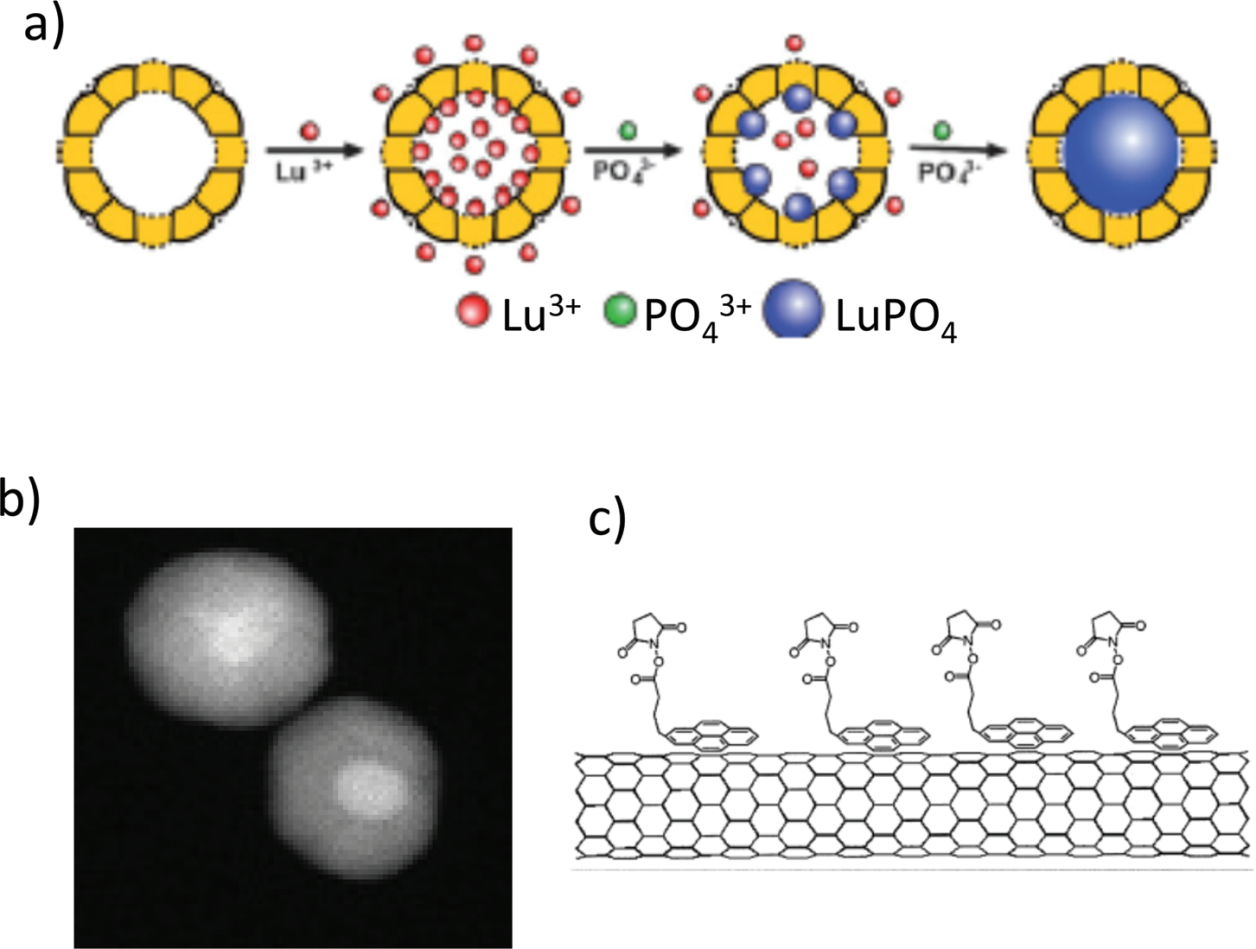
Three examples of nano-objects for which XPS analysis played an important role in understanding or verifying the nature of the objects as synthesized or after functionalization: (a) confirmation of the nature and chemistry of LuPO_4_ particles which were formed inside an apoferritin template (Ref. 55), (b) the presence of Au cores in 20 nm Ag particles (Ref. 32), and (c) the functionalization of carbon nanotubes (Ref. 56).

Although other surface sensitive analysis methods are useful for specific information,^44,53^ we frequently apply XPS as part of routine nano-object characterization as a quality check to make sure there are no compositional or contamination surprises and to quantitatively verify particle composition or functionalization (Fig. 3).^10,54–56^ In addition, we can use XPS quantitatively to estimate the thicknesses of surface coating on nanoparticles as an element of quality control and coating verification.^57–61^

Multiple approaches could be useful to directly or indirectly provide a researcher or engineer some degree of confidence that the surfaces of a set of nano-objects have the desired or expected properties.^10,53^ Whatever approaches are used should be reported as part of the provenance information.

### C. Nanoparticles are dynamic; they change with time, handling, and environmental conditions

The recognition that characteristics of nano-objects are also acquired or altered during storage, handling, and when their environment is altered highlights the importance of including information about the synthesis, processing, and handling history of nano-objects as provenance information in a material data record. There may be multiple stages in the lifetime of a set of particles with different types of characterization and handling for each. They might have a set of properties or characteristics as made, after some storage or handling, or they might be functionalized for a particular purpose giving rise to a different set of properties. The functionalized particles might then be dispersed in biological media for cellular studies again altering the particles by changing the environment. The handling, preparation, and any analysis results at each stage should become elements in the material data record.

Several common or in some cases required process steps can induce changes in nanoparticles samples or become a source of uncontrolled variability including:

(1) Material drying, heating, resuspension, or sonication: Although many particle characteristics, including size, for fresh solution synthesized six-line ferrihydrite and dried versions of the particles are similar, the reductive dissolution rates differed significantly.^62^ Details related to processing of particles such as drying, resuspension, sonication, dialysis, and heating can significantly impact particle properties and should be recorded and added to the material data record.
(2) Handling, shipping, and storage: There are many reports that purchased materials did not have the advertised/desired properties when received.^11^ This may be because changes ocurred between the time the particles were made and characterized or that measurements were made on different particles from the same or a different batch of material. Handling, time, shipping conditions, and other factors may cause material to change. It is important to record when measurements were made, especially relative to dates and duration of shipping, if the measurements were carried out on the same batch of material (or another representative batch), when a container was opened, and if other tests were conducted to generally confirm particle characteristics. Using purchased or shipped particles without any verification of properties is unwise. Even ideally made and well understood and characterized particles will have some type of shelf life, and some type of appropriate verification of consistent properties is needed before biological studies.
(3) Processing, functionalization, media exposure: Nano-objects may be processed or treated in various ways before use or application. The history of any processing for modification, along with information related to characterization before and after the processing, is an important component of provenance information. It is generally recognized that dispersion in biological media may result in removal of molecules initially on the particle surface, and in many cases, new molecular layers will form, resulting in a media modified surface. When such changes occur, a biological system will see and react with this modified surface, not the one initially on the particles.^63,64^ In particular, biological fluids generally are rich in lipid and protein components which adsorb particle surfaces, a process that has been well documented to occur in a surface-selective and size-dependent manner.^65^ The potential for the corona to modulate particle biokinetics and biological activity has been the subject of much study and several previous reviews.^63,66,67^

Our experience dispersing Ag nanoparticles in biological media demonstrates how details of particle processing steps can make significant or subtle differences in the nature of the particles as would be seen by a biological system. Particles often need to be dispersed in some type of media for surface modification, delivery to biological systems, or for other types of application. Previous work demonstrated that the dispersion of nanoparticles in cell culture media for *in vitro* testing could be enhanced by including serum in the media.^68^ Pre-exposing iron oxide particles to serum before adding to the cell culture media had been found to minimize particle agglomeration. Therefore, the same procedure was then applied to 20 nm Ag nanoparticles for *in vitro* testing.^32^ As described below additional tests were conducted, using the methods described by Munusamy *et al*.,^32^ regarding the impact of the serum addition to the stability of the Ag nanoparticles. Unlike the iron oxide particles initially studied, dissolution can be important for Ag particles and dissolved Ag contributes to the toxicity according to some studies. The specific particles examined have been characterized in considerable detail^32,58,69^ and used in several toxicology studies.^32,70–72^

We had three questions regarding the impact of the serum on the Ag particles:

(i) Would the serum alter the dissolution?
(ii) If the serum enhanced dissolution, how sensitive would the rate of dissolution be to the amount of serum?
(iii) How sensitive might the dissolution be to the way particles were added to a cell culture media serum mixture?

**Fig. 4.**
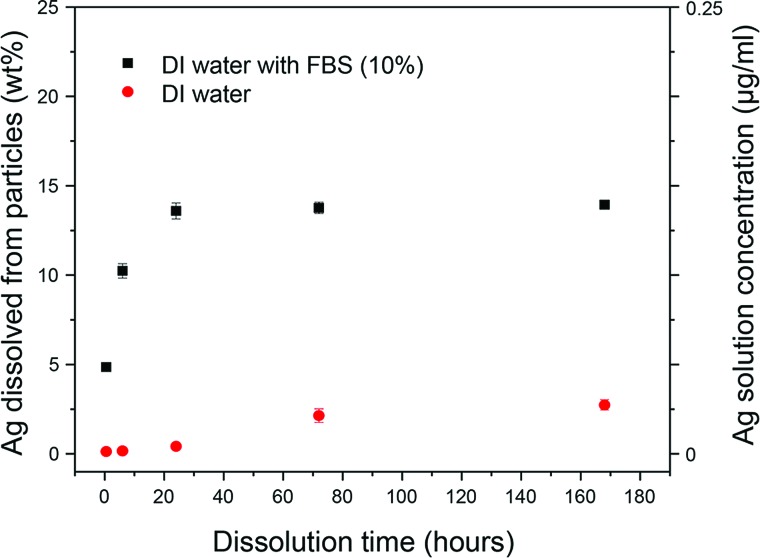
Weight percent of Ag dissolved from suspensions of 20 nm Ag nanoparticles (10 *μ*g/ml) in DI water and DI water + 10% FBS at room temperature as a function of time showing the significant enhancement of FBS on Ag dissolution. The right axis shows the concentration of dissolved Ag in the solution.

To test the impact of fetal bovine serum (FBS) on dissolution [question (i)], we examined the dissolution of particles using the procedures described in Ref. 32 in both deionized (DI) water and in DI water plus 10% FBS. As shown in Fig. 4, the addition of the FBS significantly enhanced the dissolution. It is relevant to add that when the particles are suspended in DI water they remain well dispersed and do not show aggregation. If the particles are added directly to the cell culture media, significant aggregation is observed. Therefore, the impact of FBS on dissolution could not be tested when dispersed in cell culture media with and without FBS. However, because the particles remain well suspended in both water and water with FBS, the impact of FBS on dissolution could be determined and was found to have a significant effect.

**Fig. 5.**
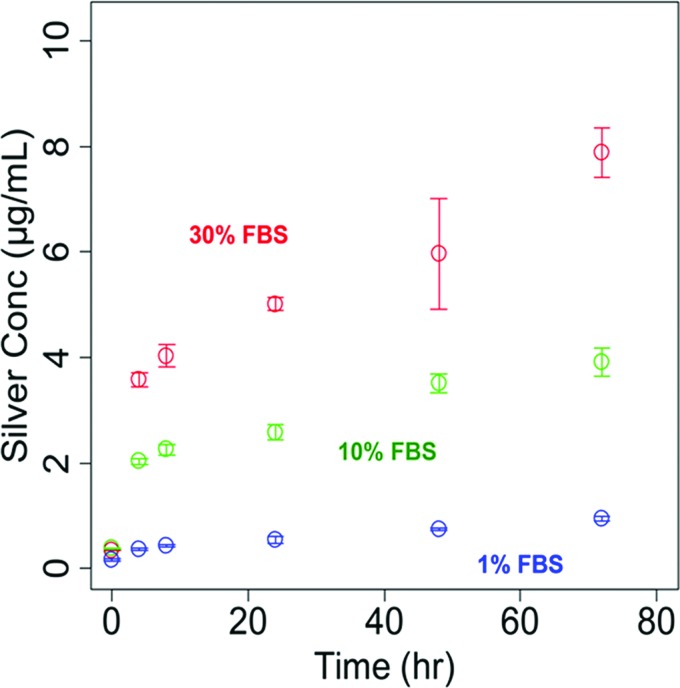
Mean (with SD) concentrations of dissolved Ag as a function of time in suspensions of 20 nm Ag nanoparticles (12.5 *μ*g/ml) in cell culture media (RPMI) with different concentrations of FBS as a function of time at 37 °C. The results show a significant enhancement of dissolution when the amount of FBS in increased.

The sensitivity of the serum enhanced dissolution to serum concentration could be tested in Rosewell Park Memorial Institute (RPMI) 1640 cell culture media. The procedure was to disperse an amount of particles in FBS and then add the particle serum mixture to the cell culture media to achieve a desired mixture of media and serum. The sensitivity of serum concentration for mixtures with 1%, 10%, and 30% [vol%] FBS is shown in Fig. 5. The dissolution rate of the Ag particles was found to be highly sensitive to the amount of serum.

**Fig. 6.**
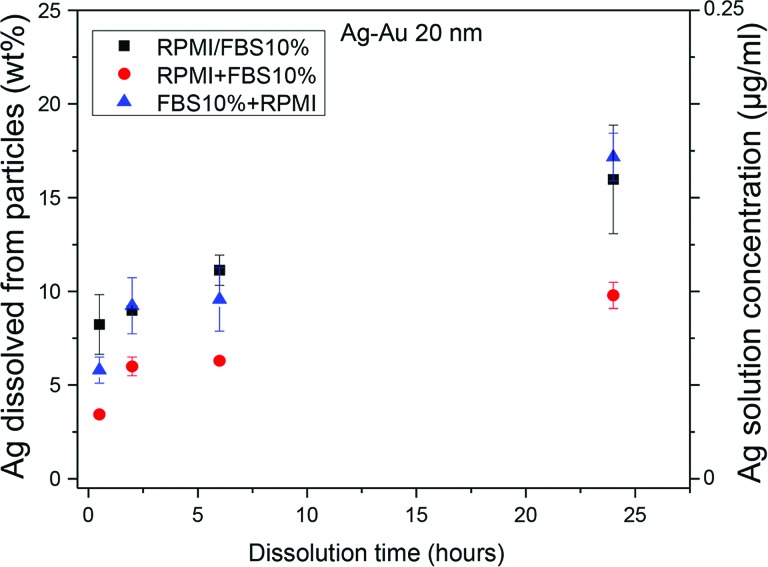
Weight percent of Ag dissolved from suspensions of 20 nm Ag nanoparticle (10 *μ*g/ml) as a function of time for particles in a mixture of cell culture media (RPMI) and FBS for different orders of mixing as described in the text. If particles were added to RPMI before adding the FBS the particles had greater aggregation and slower dissolution. The right axis shows the concentration of dissolved Ag in the solution.

For the final test (iii) the Ag nanoparticles dispersed in a mixture of cell culture media with 10% serum in three different ways: (1) particles were added first to the serum and then to the cell culture mixture (FBS 10%+RMPI); (2) particles were added to the mixed solution (RPMI/FBS10%); and (3) particles were first added to the cell culture media and then the serum was added (RPMI+FBS10%). As indicated in Fig. 6, the order of processing impacts the dissolution rate of Ag nanoparticles in the media. Based on dynamic light scattering, the hydrodynamic sizes of the nanoparticles also varied from ≈35 nm for process (a), ≈85 nm for process (b), and ≈280 nm for process (c).

There are three main messages from these measurements: (1) FBS significantly alters the dissolution of Ag nanoparticles; (2) the amount of FBS and order of mixing alters the rate of dissolution; (3) procedural details can influence effective particle size, the rate of dissolution, and the types of particles that would be observed by a biological system. Therefore, it is important to report in detail the steps taken during processing and/or to conduct some type of verification step to ensure that any small changes in processing have little consequence for the measurements to be conducted.

An interlaboratory comparison conducted by Belsey *et al*.^73^ demonstrated the challenges associated with the movement of material from solution for XPS or other surface analysis. Samples prepared using a well-defined protocol (conducted by the lead laboratory) followed by analysis in multiple labs (using protocols from the participating laboratories) produced relatively consistent data. However, when the sample preparation was done using the procedures of the participating laboratories there was wide scatter in the data. Small changes in the nature of the deposition method, the nature and cleanliness of the substrate material, and details of the drying conditions can each impact the nature of the sample produced for surface analysis.

*Analysis preparation related provenance information*: In many cases, nanoparticles are not initially in the form needed for some type of analysis or application. Powdered material may need to be dispersed in solution for DLS or zeta potential measurements.^74–76^ Particles already in solution may require dilution or concentration for some types of measurements or cleaning and drying for others.^52,57,77,78^ Because there is significant potential for changing particle coatings, surface potential, and aggregation during any of these processes, the processing steps should be recorded and reported as provenance information to be included in the sample record with sufficient detail to allow others to repeat or assess the impact of processing steps. It is also useful to conduct and report reproducibility and efforts to verify the impacts of any cleaning or depositions process. Such documentation might be prepared for each sample or by referencing previously described procedures or publications, having followed the procedures with care and verified both the consistency and reasonableness of the results.

### D. Recognizing the issues makes success possible

Although preparing and delivering reproducible batches of nanoparticles for specific purposes can be challenging, many research teams have found ways to be successful. Recognizing that reproducibility and reliability are critical issues, especially for nanomaterials, and understanding some of the physical and chemically related processes that can cause problems helps suggest ways to improve material reliability. If materials used in a study are not reproducible, work following from the study will likely be unreliable and not reproducible. Although the issues related to nanomaterials seem more endemic than for most other materials, the problem materials' reproducibility is sufficiently general and widespread that a virtual issue of the journal Chemistry of Materials was assembled to deal with matters of materials reproducibility “Best Practices for Reporting the Properties of Materials and Devices.”^79^

## III. SUMMARY AND CONCLUSIONS

Relevant provenance information about batches of nanoparticles can provide a useful tool for identifying and minimizing some of the sources of different behaviors of supposedly similar nanoparticles in biological studies.

A wide variety of reports and editorials indicate that there are significant challenges in creating and delivering well characterized nano-objects in a consistent and uniform matter for both study and application. Issues range from differences, subtle and otherwise, in nano-object synthesis, in how nano-objects are characterized, handled, stored, processed, and prepared for analysis. It is useful to recognize that in many circumstances nano-objects are not stable in their environment. Therefore, it can be important to understand in detail how a set of particles were synthesized, their initial properties (intrinsic or native properties), and what has happened to the particles since the time of synthesis and characterization.

The information that needs to be associated with a set of nano-objects to reliably trace their properties and behaviors since synthesis may be usefully identified as provenance information and this should be included in some type of materials associated data record. Other researchers are developing data recording protocols to enhance the ability to extract useful information from collections of curated data on nano-objects.

Based on our experience, the provenance information should include (1) detailed records regarding sample synthesis; (2) results of characterization, including any sample handling needed for the characterization; (3) history of the material including important dates and times such as date of synthesis, arrival in a laboratory, when containers were opened; (4) information about storage conditions, time, and containers; and (5) summary of any additional processing before later use or analysis. An ISO standard (ISO TS 20579-4)^80^ is being prepared to address information needed regarding preparation of samples for surface analysis to be added to provenance information for a material.

Although provenance information and an appropriate data record can assist delivery of reproducible particles, such data only assists the process. The ultimate test for a biological application is consistent biological response. Provenance information may help in dealing with identified issues, but positive and negative control measurements and other validation tests can help verify nano-object consistency in the environments of actual use or application.
